# Antidiarrheal and Antibacterial Activities of *Calpurnia aurea*: Benth Seed Different Extracts

**DOI:** 10.1155/2022/9582687

**Published:** 2022-08-31

**Authors:** Achenef Bogale, Haile Alemayehu, Teshome Nedi, Ephrem Engidawork

**Affiliations:** ^1^Department of Pharmacy, College of Health Sciences, Debre Tabore University, P.O. Box 272, Debre Tabore, Ethiopia; ^2^Aklilu Lemma Institute of Pathobiology, Addis Ababa University, Addis Ababa, Ethiopia; ^3^Department of Pharmacology and Clinical Pharmacy, School of Pharmacy, College of Health Sciences, Addis Ababa University, P.O. Box 1176, Addis Ababa, Ethiopia

## Abstract

**Background:**

*Calpurnia aurea* is believed to have antidiarrheal potential but with limited scientific evidence. This study aimed investigating antidiarrheal and antibacterial activity of aqueous and 80% methanol seed extracts of the plant in mice and selected diarrhea-causing bacterial strains, respectively.

**Methods:**

Castor oil-induced diarrhea, prostaglandin-induced enteropooling, and castor oil-induced charcoal meal test models in mice of either sex using three dose levels (60, 120, and 240 mg/kg) were applied to evaluate antidiarrheal activity. Parameters, including onset, number, wet stool weight, weight and volume of secretion, and intestinal motility, were taken into consideration. The antibacterial activity was assessed on *Shigella soni, Salmonella typhimurium, Escherichia coli, Staphylococcus aureus*, and *Pseudomonas aeruginosa* using disk diffusion and microdilution techniques.

**Results:**

Compared to controls, pretreatment of mice at the graded dose (60, 120, and 240 mg/kg) resulted in a significant (*p* < 0.05) drop in frequency of wet stools and watery content of diarrhea as well as in delaying onset of diarrhea. Both extracts exhibited inhibition of diarrhea in a dose-dependent manner in all models used. The extracts also showed significant (*p* < 0.05) reduction in intestinal motility in castor oil-induced models. Both extracts showed a marginal activity against the selected bacterial strains; a better effect was seen with 80% methanol seed extract.

**Conclusion:**

Both extracts of the plant have beneficial effect in controlling diarrhea. This finding supports the use of the plant as a traditional antidiarrheal remedy.

## 1. Background

All through human history, diarrheal diseases have been a major health problem. In the past, it was often deadly and threatening large populations [1]. Even though possible to treat and prevent, diarrhea still continued to take the lives of under five children worldwide, and it is the second leading cause of mortality for this age group next to respiratory infections. Children killed by diarrhea are even more than those killed by AIDS, malaria, measles, injuries, and all other postneonatal conditions combined [2]. Today, despite the successful interventions such as oral and intravenous rehydration therapy, diarrheal diseases remain one of the leading causes of morbidity and mortality in the world. In Africa, including Ethiopia, each child on average suffers from five episodes of diarrhea per year [3]. Even though global child mortality rate decline to 37 per thousand births from 1990 to 2020, Africa's child mortality rate remains not less than 74 per thousand births during this period [4].

Natural products have a unique chemical diversity, which enable them to have a varied biological activities and drug-like properties [5]. Across the globe, there are various herbal plants that possess antidiarrheal activity. Several bioactive compounds such as tannins, alkaloids, saponins, flavonoids, steroids, and terpenoids could be take the credit. [5, 6]. *Calpurnia aurea* is a flowering plant within the family of Fabaceae. The genus embraces shrubs or small trees in or along the margin of forests in many parts of Ethiopia [7]. It is widely distributed in Africa from Cape Province to Eritrea. The Southern part of India is also additional habitat for the plant [7, 8].

In different parts of Ethiopia, *C. aurea* is claimed to treat different type of disease conditions, such as amoebic dysentery, diarrhea, syphilis, tapeworm, leishmaniasis, trachoma, wound, *Tinea capitis*, scabies, elephantiasis, and diverse swellings in humans [9–12]. *C. aurea* is a well-studied plant. It is found to have antioxidant [13], antihypertensive [14], antibacterial [6], antimalarial [15], and antidiabetic activity [16].

Methanol (80%) leaf extract of *C. aurea* has been reported to have antibacterial activity against clinical isolates of *Salmonella typhi*, *Salmonella typhimurium*, *Salmonella paratyphi*, *Shigella* species, *Pseudomonas aeruginosa*, *Staphylococcus aureus*, and *Escherichia coli*. Not only this, the *C. aurea* extract also reported to have *in vivo* antidiarrheal effect in castor oil-induced diarrheal model [6]. As a preliminary phytochemical screening studies, *C. aurea* is endowed with different type of secondary metabolites such as alkaloids, flavonoids, saponins, phenolic, tannins, terpenoids, cardiac glycosides, and steroids, with alkaloids and tannins are being more concentrated in seeds than leaves [17]. Although the seeds of *C. aurea*, like that of the leaf and root, is claimed to treat diarrhea in Ethiopia [10, 11], it is not still scientifically investigated. Therefore, the contemporary study aimed to investigate the *in vitro* antibacterial and *in vivo* antidiarrheal activities of aqueous and 80% methanol seed extract of *C. aurea.*

## 2. Methods

### 2.1. Drugs and Chemicals

Castor oil (Amman Pharmaceutical Industries, Jordan), activated charcoal (Acuro Organics Ltd, India), loperamide hydrochloride (Medochemie Ltd, Cyprus), misoprostol (Mylan Laboratories Ltd, India), distilled water (Department of Pharmaceutics and Social Pharmacy of Addis Ababa University, Ethiopia), methanol (Blulux, India), petroleum ether (Carlo Erba Regents S.A.S, Italy), McFarland standard (Remel, Lenexa Kansas 66215, USA), brain heart infusion (BHI) (Difco Laboratories, Detroit Michigan, USA), ciprofloxacin disk 5 mcg (Ecton Dickinson Pty Ltd, Australia), Muler Hinton agar (Himedia laboratories Pvt Ltd, India), and Muler Hinton broth (Himedia laboratories Pvt Ltd, India) were used in this study. All chemicals and solvents were of analytical grade.

### 2.2. Plant Materials

The dried ripe seeds of *Calpurnia aurea* were collected during early morning time of autumn from Addis Zemen, which is located at a latitude and longitude of (12°07′N37°47′E) and an elevation of 1975 meters above sea level in south Gondar zone of Amhara region. Following collection, a taxonomist (Mr. Melaku Wondafrash) identified and authenticated the plant, and a voucher specimen (001AB) was deposited at the National Herbarium, College of Natural and Computational Sciences, Addis Ababa University for future reference.

### 2.3. Experimental Animals

Adult mice of both sexes (20–30 g) aged 6–8 weeks were obtained from animal house unit of School of Pharmacy, College of Health Sciences, Addis Ababa University. Polypropylene cages (6–10 animals per cage) were used for animal housing under standard environmental conditions on a 12 h light-dark cycle with access to pellet food and water *ad libitum*. Acclimatization of animals for the environment for about a week was used before beginning the actual experiment. All experiments were conducted during the light period. All the protocols were approved by the School of Pharmacy Ethics Committee (ERB/SOP/180A/13/2020) and were conducted according to the guideline for the care and use of laboratory animals [18, 19].

### 2.4. Test Strains

*Salmonella typhimurium* (ATCC13331), *Shigella soni* (ATCC12022), *Pseudomonas aeruginosa* (ATCC27853), *Staphylococcus aureus* (ATCC25923), and *Escherichia coli* (ATCC25922) were obtained from Akililu Lemma Institute of Pathobiology, Addis Ababa University. All are standard strains.

### 2.5. Extraction and Preparation of the Plant Material

First, ripe and dried seeds of *C. aurea* were washed using distilled water to remove contaminants. Second, the seeds were grinded and powdered coarsely using mortar and pestle before extraction.

### 2.6. Preparation of 80% Methanol Extract

Eighty percent of methanol was used to extract *C. aurea* seeds powder were extracted using 80% methanol through maceration technique. One hundred gram of coarse powder of the plant material was macerated with about 500 ml of the solvent at room temperature for 72 h and was shaken continuously on a horizontal orbit shaker. Then, the mixture was filtered through a muslin cloth and Whatman No. 1 filter paper. The leftover marcs were remacerated twice using equal volume of solvent to extensively extract the plant material. After this, the methanol was removed from the extract using a Rotavapor at 40°C under reduced pressure. Then, a lyophilizer was used to remove water and the resulting dry hydroalcoholic extract of the plant was weighed, and percentage yield was found to be 15.5%. Finally, the dried extracts of the plant were kept at −20°C and was reconstituted with distilled water for oral administration.

### 2.7. Preparation of Aqueous Plant Extract

The plant material was extracted using cold maceration technique. One hundred gram of the coarse powder was soaked in an Erlenmeyer flask with 1 L of distilled water and then placed on a shaker tuned at 120 rpm with occasional shaking for 72 h at room temperature. The extract was filtered first using a muslin cloth and then Whatman No-1 filter paper and the marc was remacerated for a second and third time by adding another fresh solvent. The filtrates were left overnight in a deep freezer and then were lyophilized using a freeze dryer. After drying, percentage yield of crude aqueous seed extract of *C. aurea* was found to be 17.5%. The dried plant extract was reconstituted with distilled water for oral administration [20].

### 2.8. Acute Toxicity Test

First, acute toxicity test was done based on the limit test recommendations of Organization for Economic Cooperation and Development (OECD) 425 Guideline [21]. First, two female mice were fasted for 4 h and then loaded with 2000 mg/kg of either of the extracts, orally. The mice were then observed for physical or behavioral changes. Since both mice died within 30 min of administration of each extract, based on OECD 425 guideline, the main test procedure was performed. The mice were observed continuously for 4 h with 30 min interval and then for 14 consecutive days with an interval of 24 h for the general signs and symptoms of toxicity such as change in food and water intake and also for mortality.

The main test procedure was performed by using a default dose progression factor of 3.2. Based on the recommendation of OECD 425 guideline, since estimate of the substance's lethality is not available, the starting dose for testing procedure was determined to be 175 mg/kg. Then by using the dose progression factor of 3.2, the next doses 550 mg/kg and 1750 mg/kg, tested successively. Each single animal was dosed in sequence at 48 h intervals. Treatment of an animal at the next dose was delayed until the survival of the previously dosed animal was confirmed.

### 2.9. Grouping and Dosing

Thirty mice (for each solvent extracts) of either sex was randomly divided into five groups of six mice each and were fasted for 18 h before the test with free access to water. Group I served as negative control and treated with 10 ml/kg normal saline. Group II serving as a positive control was treated with loperamide (3 mg/kg). Group III, IV, and V were given 60, 120, and 240 mg/kg of methanol and aqueous extracts of the *C. aurea* seeds orally. For enteropooling model, Misoprostol (a PGE_2_ analog) was used to induce diarrhea, and castor oil is replaced in the remaining models for the same function. All doses were administered orally, and maximum volume administered was 10 ml/kg.

### 2.10. Determination of Antidiarrheal Activity

#### 2.10.1. Castor Oil-Induced Diarrhea

The castor oil-induced diarrheal model followed by Umer et al. was used to evaluate antidiarrheal activity of *C. aurea* in mice [6]. Forty-eight mice of either sex were randomly divided into eight groups of six mice each and were used after overnight fasting. The plant extract was given to mice as described in grouping and dosing section. One hour latter, 0.5 ml of castor oil was administered to each mouse orally. The mice were then housed separately in transparent metabolic cages, the bottom of which was covered with white sheet of paper to make observation of consistency and numbering of fecal droppings easy. Since the papers were changed every hour, counting and checking of stool consistency was not difficult. Diarrhea was graded as follows: normal pelleted feces (0), discrete soft-formed feces (1), soft-formed feces (2), soft watery stool (3), and watery stool with little solid matter (4) [22].

The animals were observed for a period of 4 h, during which the onset of diarrhea, the number and weight of both dry, and wet stools excreted by the animals were recorded and compared with the control for assessing the antidiarrheal activity. The onset was measured as the time interval in minutes between the administration of castor oil and the appearance of the first diarrheal stool. The total number of diarrheal feces of the control group was considered 100%, and percentage of diarrheal inhibition for wet and watery content of feces was determined using the following formula:

(1) $$\begin{array}{c} \%\text{ inhibition}=\frac{\text{AWFC}-\text{AWFT}}{\text{AWFC}}\times 100, \end{array}$$

where AWFC = average weight of feces in the control group and AWFT = average weight of feces in the test group [22].

### 2.11. Enteropooling Test

Forty-eight mice of either sex were randomly divided into eight groups of six mice each and were used after overnight fasting [21]. Mice were dosed as described in grouping and dosing section. Misoprostol was administered 1 h after dosing. Then, 1 h after administration of 100 *μ*g/kg of misoprostol, all mice were sacrificed by cervical dislocation, and the small intestine accumulated with fluid was ligated both at the pyloric sphincter and at the ileocecal junctions and dissected out. The tied intestine was weighed (*m*_1_); its content emptied into a graduated cylinder and its volume was measured. The emptied intestine was then weighed (*m*_0_), and the difference between the empty and intact intestine was used to calculate percentage inhibition of intestinal secretion relative to control group using the following formula:

(2) $$\begin{array}{c} \%\text{ inhibition}=\frac{\left(A-B\right)}{A}\times 100, \end{array}$$

where *A* = average volume or weight of intestine in control group and *B* = average volume or weight of intestine in test groups [22].

### 2.12. Charcoal Meal Test in Normal Mice

Experimental procedure described by Bahekar and Kale [23] was used with slight modification. Fort-eight mice (control group remain the same for both extract) of either sex were randomly divided into eight groups of six mice each and fasted for 18 h prior to the test but were allowed free access to water. One hour after dosing, each animal was given 1 ml of freshly prepared charcoal meal (10% active charcoal suspension in 2% tween 80) orally. One hour after charcoal administration, animals were sacrificed. The abdomen was opened, and the small intestine from the pylorus to caecum was taken out. The distance travelled by the charcoal meal in the intestine, from the pylorus to the caecum, was measured and expressed as the percentage of distance covered using the following formula:

(3) $$\begin{array}{c} \%\text{ Transit inhibition}=\frac{T_{0}-T_{1}}{T_{0}}\times 100. \end{array}$$

*T*_0_ = total length of intestine, *T*_1_ = distance travelled by charcoal in intestine,

(4) $$\begin{array}{c} \%\text{ inhibition}=\frac{\text{mean of distan ce traveled by mar ker of }\left(\text{control}-\text{test}\right)\text{group}}{\text{mean of dis tan ce traveled by mar ker of control group}}\times 100. \end{array}$$

### 2.13. Charcoal Meal Test following Induction of Diarrhea

The effect of the *C. aurea* seed extract on gastrointestinal motility was evaluated as described by Umer et al. [6] with some modification. Forty-eight mice of either sex were randomly divided into eight groups of six mice each and were used after overnight fasting [22]. After one hour of dosing the plant extract, per oral administration of 0.5 ml of castor oil was done. After 1 h of castor oil administration, all animals received 1 ml of charcoal meal marker (10% charcoal suspension in 2% tween 80) orally, and all of them were sacrificed after 30 min of marker administration. The small intestine was dissected out, and the distance travelled by charcoal meal from the pylorus to caecum was measured and expressed as a percentage of the total distance of the small intestine. The intestine of each mouse was immersed in formalin to arrest peristalsis and then washed in clean tap water before measuring the distance travelled by the charcoal meal. Charcoal movement was assessed as a peristaltic index (PI) as follows:

(5) $$\begin{array}{c} \text{PI}=\frac{A}{B}\times 100, \end{array}$$

where *A* = distance travelled by charcoal meal and *B* = length of full intestine. Percentage inhibition is also calculated as follows:

(6) $$\begin{array}{c} \text{Percentage inhibition}=\frac{\text{APIC}-\text{APIT}}{\text{APIC}}\times 100, \end{array}$$

where APIC = average PI of control and APIT = average PI of test group [22].

### 2.14. *In vivo* Antidiarrheal Index (ADI)

ADI of treated groups was determined using data from castor oil-induced diarrhea, enteropooling, and gastrointestinal motility tests using the formula described by Umer et al. [6].

(7) $$\begin{array}{c} \text{ADI }in\;vivo=\sqrt[{3}]{\text{DDT }\times \text{ GMT }\times \text{ IFA}}, \end{array}$$

where DDT is the delay in defecation time or diarrheal onset (as % of control), GMT is the gastrointestinal motility by charcoal travel reduction (as % of control), IFA is the reduction in the intestinal fluid accumulation (as % of control),

(8) $$\begin{array}{c} \text{DDT}=\frac{\text{Onset of diarrhea in minute of the}\left(\text{test}-\text{negative control}\right)\text{groupy}}{\text{Onset of diarrhea in minute of the negative control groupx}}\times 100\text{,} \end{array}$$

(9) $$\begin{array}{c} \text{GMT}=\frac{\text{Distance travelled by the charcoal marker of the}\left(\text{negative control}-\text{test}\right)\text{group}}{\text{Distance travelled by the charcoal marker in the negative control group}}\times 100, \end{array}$$

(10) $$\begin{array}{c} \text{IFA}=\frac{\text{Mean weight of wet stools of }\left(\text{negative control }-\text{treated}\right)\text{group}}{\text{Mean weight of wet stools of negative control group}}\times 100. \end{array}$$

### 2.15. Determination of Antibacterial Activity

#### 2.15.1. Inoculum Preparation and Standardization

The bacteria were selected based on availability and consideringthe likely bacterial strains that can cause diarrhea for which the experimental plant is indicated traditionally. Nutrient agar was prepared following the manufacturer's protocol. After cooling the media to about 45°C, it was poured to a prelabelled sterile Petri dishes aseptically and allowed time for congealing of the agar. Thestandard pathogenic bacteria were then inoculated and spread on the prepared agar using inoculating wire loop following aseptic condition and incubated for 24 h at 37°C.

The inoculum of each of bacterium was prepared and standardized by following the guideline of Clinical and Laboratory Standard Institute [24]. The bacterial suspension in a broth was prepared by using the appropriate methods. After preparing nutrient broth in distilled water, 5 ml of the broth was transferred to test tubes and autoclaved. Isolated colonies of the same morphological type of each bacterium from 3–5 wells were picked up by wire loop from fresh agar plates of bacterial culture and aseptically transferred into prelabelled test tubes containing the sterile nutrient broth and incubated for about 6 h. The turbidity of the inoculum tube was adjusted visually by adding either bacterial colonies or sterile normal saline solution to that of the already prepared 0.5 McFarland standard, which is assumed to contain a bacterial concentration of 1 × 10^8^ CFU/ml. The adjustment and comparison of turbidity of inoculum tube and that of 0.5 McFarland standard was performed by visually observing them with naked eye against a 0.5 McFarland turbidity equivalence standard card with white background and contrasting black lines in the presence of adequate light.

### 2.16. Agar Well Diffusion

Agar well diffusion method was used to determine antibacterial activity. Diluted inoculums (0.1 mL) of test organism (10^8^ A CFU/mL) were spread on Muller-Hinton agar plates. Wells of 6 mm diameter were punched into the agar medium with sterile cork-borer under aseptic conditions and was filled with a serial dilution of 50 *μ*l of plant extract starting from 1000 mg/ml, solvent blank and standard antibiotic (ciprofloxacin 5 *μ*g). The plate was kept at room temperature for 2 h for diffusion and then incubated for 24 h at 37°C. Antibacterial activity was evaluated by measuring the zone of inhibition against the test organisms [25]. Ciprofloxacin (5 *μ*g disc) was used as a reference standard, and distilled water was used as a control. The growth was compared with the reference as well as the control. Each experiment was repeated three times.

### 2.17. Determination of Minimum Inhibitory Concentration (MIC)

The extracts of both solvents that showed antibacterial activity by agar well diffusion method was subjected to serial microbroth dilution technique to determine MIC as described by Mukherjee et al. [26]. Serial dilutions were prepared from 1000 mg/ml of the plant extract using distilled water to make 1000, 500, 250, 125, 62.5, 31.25, and 15.625 mg/ml. The wells were inoculated with 0.1 mL aliquot of test organisms (10^8^ CFU/mL) having serial dilutions of the extract (50 *μ*l, each). The microplate was incubated at 37°C ± 1°C for 24 h. Dilution of the extract corresponding to respective test organism showing no visible growth was considered as MIC.

### 2.18. Determination of Minimum Bactericidal Concentration (MBC)

The MBC is defined as the lowest concentration where no bacterial growth is observed. This was determined by aseptically subculturing the contents of wells from the MIC results for individual bacterium to antimicrobial free agar as described by Mukherjee et al. [25]. In this technique, the contents of all wells containing a concentration of test material above the MIC value from each triplicate, in the MIC determination test, were streaked using a sterile wire loop on Muller-Hinton agar aseptically and incubated at 37°C for 24 h. The lowest concentration of each extract, which showed no bacterial growth after incubation, was observed for each triplicate and noted as the MBC. The average value was taken for the MBC of test material against each bacterium.

### 2.19. Statistical Analysis

Results are expressed as mean ± standard error of the mean (SEM). The results were analyzed using Statistical Package for Social Sciences (SPSS), version 20 software, and statistical significance was determined by one-way analysis of variance (ANOVA) followed by Tukey Kramer post hoc test. *p*-value of less than 0.05 was considered as statistically significant.

## 3. Results

### 3.1. Acute Toxicity Test

Aqueous and 80% methanol seed extracts of *C. aurea* were studied for acute toxicity at a dose of 2000 mg/kg by oral route. However, mice that received either of the extract died 30 min following administration of the extract. Based on OECD 425 guideline, the main test procedure was performed, and dose estimator of both extracts was determined to be 1200 mg/kg. Accordingly, the graded dose of 60, 120, and 240 mg/kg of each extract was used for the experiment.

### 3.2. Effect of the Aqueous and 80% Methanol Seed Extracts on Castor Oil-Induced Diarrhea

During the four hours observation period, all mice in the control group had either wet stool or watery diarrhea. In case of water extract, pretreatment of mice at the dose of 60 (*p* < 0.05), 120 (*p* < 0.05), and 240 mg/kg (*p* < 0.001) significantly delayed the onset of diarrhea. The 80% methanol extract, however, produced a significant (*p* < 0.001) effect on the onset only at the higher dose of 240 mg/kg. Both extracts at all dose levels were able to significantly (*p* < 0.001) reduce the frequency of diarrhea (Table 1). Moreover, both extracts delayed the onset of diarrhea (*R*^2^ = 1.00 for methanol extract and 0.946 for aqueous extract) and reduced the frequency of defecation (*R*^2^ = 0.80 for aqueous extract and 0.893 for methanol extract) in a dose-dependent manner as compared to the negative control. For each extract, the percentage of inhibition for both total weight of wet diarrhea and watery content of diarrhea relative to negative controls revealed that, except for the lower dose of the aqueous extract, all the other doses of both extracts produced a significant (*p* < 0.05) decrease in both total weight and watery content of diarrhea compared to controls. Otherwise, there was no detectable difference between standard and extracts as well as among various doses of both extracts (Table 1).

### 3.3. Effect on Prostaglandin-Induced Enteropooling

**Figure 1:**
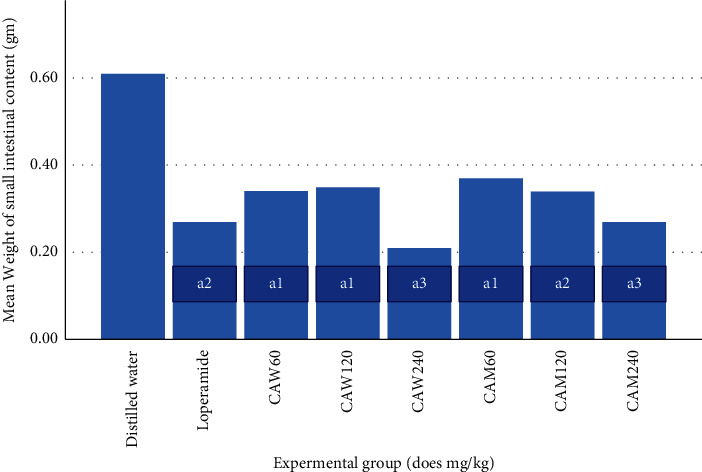
Effects of the aqueous and 80% methanol seed extracts of *C. aurea* on prostaglandin-induced enteropooling (weight of intestinal content) in mice. Values are expressed as mean ± S.E.M (*n* = 6). Comparison was made among different groups: ^a^compared to control; ^e^ to 240 mg/kg aqueous ^1^*p* < 0.05, ^2^*p* < 0.01, ^3^*p* < 0.001. DW = distilled water, L = loperamide, CAW = *C. aurea* water extract, and CAM = *C. aurea* 80% methanol extract.

**Figure 2:**
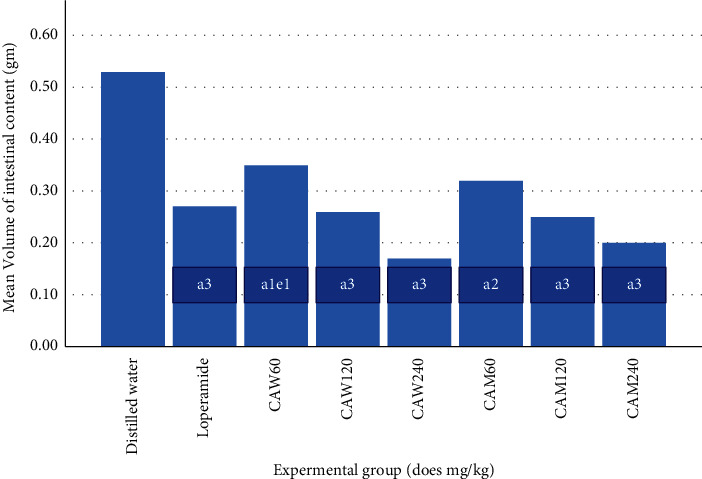
Effects of the aqueous and 80% methanol seed extracts of *C. aurea* on prostaglandin-induced enteropooling (volume of intestinal content) in mice. Values are expressed as mean ± S.E.M (n = 6). Comparison was made among different groups: ^a^compared to control; ^e^ to 240 mg/kg aqueous ^1^*p* < 0.05, ^2^*p* < 0.01, ^3^*p* < 0.001. DW = distilled water, L = loperamide, CAW = *C. aurea* water extract, and CAM = *C. aurea* 80% methanol extract.

Percentage inhibition in intestinal fluid accumulation was 33.96, 50.94, and 60.92% for water extract and 39.62, 52.83, and 62.26% for 80% methanol extract at 60, 120, and 240 mg/kg doses, respectively (Figures 1 and 2). In both cases, the antisecretory effect of the plant increased with dose (*R*^2^ = 0.96 for aqueous extract and, 0.92 for 80% methanol extract). Both aqueous and methanol seed extracts of C*. aurea* showed a significant reduction (*p* < 0.05) in average weight as well as volume of small intestine content at all doses. Furthermore, 240 mg/kg dose of water extract of *C. aurea* seed reduced mean volume of intestinal content significantly as compared to that of 60 mg/kg. However, there was no statistically significant difference in terms of volume of intestinal fluid and weight of intestinal contents when all doses of both extracts were compared with the standard drug.

### 3.4. Effect on Castor Oil-Induced Gastrointestinal Propulsion

Both aqueous and 80% methanol seed extracts of *C. aurea* exhibited a statistically significant (*p* < 0.001) antimotility effect against castor oil-induced diarrhea compared to negative controls (Table 2). Both extracts significantly inhibited the intestinal transit of charcoal meal at all doses, with the higher dose exhibiting the maximum effect (63.4% for aqueous 61.1% for 80% methanol extract). The effect was dose dependent, *R*^2^ being 0.733 for aqueous extract and 0.861 for 80% methanol extract. The standard drug produced a significantly higher effect compared to controls (*p* < 0.001) as well as lower dose (*p* < 0.001) of both extracts.

### 3.5. Effect on Normal Gastrointestinal Transit in Mice

Both aqueous and 80% methanol seed extracts of *C. aurea* tended to decrease the intestinal transit of the charcoal meal compared to the control group (Table 3); however, the change did not reach statistical significance. By contrast, the inhibition obtained with the standard drug was significantly greater (29.6%, *p* < 0.05) than the control group. The effect of the extract was dose dependent (*R*^2^ = 0.60 and *R*^2^ = 0.81 for water and 80% methanol extract, respectively), indicating the risk of constipation with increasing dose.

### 3.6. *In vivo* Antidiarrheal Index

The antidiarrheal index for the different doses of the extracts is presented in Table 4. The index seems comparable for both extracts and showed significant (*p* < 0.05) pooled effect.

### 3.7. Antibacterial Activity

Both aqueous and 80% methanol seed extracts of *C. aurea* showed marginal antibacterial activity against the selected strains, except *P. aeruginosa*. The 80% methanol seed extract appeared to be somewhat better than the aqueous seed extract (Tables 5 and 6). Among the selected bacterial strains, *S. typhimurium* was the most sensitive one in terms of MIC and MBC. Its growth was inhibited at a concentration of 250 mg/ml and completely killed at a concentration of 500 mg/ml. In terms of zone of inhibition, however, *E. coli* showed better sensitivity. Although the test was performed up to a concentration of 7.8 mg/ml for both extracts, the smallest dose with antibacterial activity against the selected strains was 125 mg/ml. The results of antibacterial activity in general indicates that the antibacterial activity of *C. aurea* is not responsible for the antidiarrheal effect.

## 4. Discussion

Evaluating the *in vivo* antidiarrheal and *in vitro* antibacterial activity of aqueous and 80% methanol seed extracts of *C. aurea* in mice and selected bacterial strains, respectively, and the probable underlying mechanism was the focus of conducting this study. The results exhibited that the plant is endowed with antidiarrheal and minimal antibacterial activity in the models used.

Castor oil causes diarrhea due to its active metabolite, ricinoleic acid [26]. In addition to castor oil, administering misoprostol is another option [22]. Using castor oil and misoprostol as diarrhea inducer makes it credible since they represent the pathophysiologic processes.

Imparted to its antimotility and antisecretory properties, the standard drug (loperamide hydrochloride) has been reported to slow down small intestine transit time [27].

In the present study, both extracts of *C. aurea* exhibited significant antidiarrheal activity by inhibiting castor oil- and prostaglandin induced-diarrhea. Although both extracts showed a significant difference from negative control, there was no apparent difference between the two extracts. This may be due to the presence of similar secondary metabolites in both extracts.

A statistically significant (*p* < 0.05) reduction in the number and weight of fecal output, not to mention, a delayed onset of diarrhea was observed in both extracts. The antidiarrheal effect of the plat was increased in a dose-dependent fashion. This infers that a better antidiarrheal effect is expected at higher dose. Other species of plants also reported to have antidiarrheal effect in a similar manner [28].

Fall down in frequency of defecation and weight of stools implies the efficacy of both aqueous and 80% methanol seed extracts of *C. aurea* as antidiarrheal agent. Traditionally claimed plants such as *Eremomastax speciosa* and *Xylocarpus granatum* showed similar finding with the current plant [29]. Castor oil induces diarrhea via inhibition of fluid and electrolyte reabsorption which increase intestinal peristalsis [30]. One of the probable mechanisms for antidiarrheal activity of *C. aurea* seed extract might be due to its ability to enable fluid and electrolyte absorption in the GI tract.

Furthermore, the delayed onset of diarrhea, inhibition of defecation and decreased number of wet feces following the administration of both seed extracts proves antidiarrheal activity of *C. aurea*. Percentage of inhibition of defecation at 240 mg/kg dose of the extract, comparable to loperamide hydrochloride, indicates that the plant has a potential antidiarrhea effect. The extracts might have exerted their antidiarrheal activity via antisecretory mechanism as evident from reduction in total number of wet feces and weight of watery content of diarrhea. Furthermore, this antidiarrheal activity might have resulted from the inhibitory activity of both extracts on PGs synthesis, NO, and platelet activating factors production [31]. Similar findings were reported in studies by Qnais et al. [32], Akindele and Adeyemi [33], and Appidi et al. [34] following the administration of aqueous leaf extracts of *Juniperus phoenicea*, *Byrsocarpus coccineus*, and *Herdmania incana*, respectively.

Reduction in weight of watery content of diarrhea is higher than that of weight of wet stool diarrhea in both extracts of *C. aurea*. From this, we can infer the probable antidiarrheal mechanism of the plant that is increasing absorption or/and decreasing secretion of fluid and electrolytes.

The study continued further and investigated antienteropooling effect of the plant using misoprostol. In this test, all tested doses of both aqueous and 80% methanol seed extracts of *C. aurea* significantly (*p* < 0.05) inhibited intestinal fluid accumulation and weight of intestinal content compared to the negative control. In this aspect, the effect of both extracts is comparable. It may be due to the presence of comparable active metabolites in both extracts. Furthermore, 240 mg/kg dose of water extract of *C. aurea* seed reduced mean volume of intestinal content significantly as compared to that of 60 mg/kg. The presence of secondary metabolites such as terpenoids, steroids, flavonoids, and tannins could be responsible for antienteropooling activity of the plant. Secondary metabolites, such as terpenoids [35], flavonoids [36], and steroids [37], have been implicated in the inhibition of PGE_2_ production [38–40]. A protein-precipitating effect of tannins in the GI mucosa [14], which makes intestinal lining resistant to chemical reaction, has been implicated for its antidiarrheal effect [41].

PG might also activate the NO pathway and induce NO-dependent GI secretion [42] through the stimulation of cAMP and cGMP concentration [43]. A growing body of evidence indicates that bioactive compounds such as terpenoids [35, 44], alkaloids [43, 45], and flavonoids [46] have a significant role in the inhibition of NO synthesis. This indicates that interruption of NO pathway could be one of the probable mechanisms of antienteropooling effect for both extracts.

Moreover, small intestine is innervated by both parasympathetic and sympathetic nervous system [47]. Parasympathetic system is responsible for intestinal secretion through acetylcholine and vasoactive intestinal peptides, whereas sympathetic system antagonizes this effect via *α*_2_ adrenergic receptors. Secondary metabolites such as flavonoids enhance *α*_2_ adrenergic receptors in the absorptive section of the GI tract [42]. Management of aquaporin water channels is implicated in controlling transepithelial fluid transport in the GI tract. Tannins were found to block these channels expressions (specifically aquaporins 2 and 3) *in vivo* and *in vitro* via downregulating protein kinase A/cAMP response element-binding protein (PKA/CREB) signal pathway [48]. All these evidences support that antisecretory effect of extracts might probably relate to the existence of flavonoids, tannins, terpenoids, and saponins and their synergistic effects. In the present study, stimulation of reabsorption and inhibition of secretion by affecting autonomic nervous system might be one of the probable mechanisms to reduce diarrhea. The antientropooling effect of the standard drug loperamide is somewhat lower than its effect on motility (Table 4). However, this is not unexpected and without reason. A study by Schiller et al. confirmed that the major antidiarrheal mechanism of action of loperamide is reduction of intestinal motility rather than proabsorptive or antisecretory effect [49].

Charcoal meal was used as a marker for antimotility test. A standard drug, loperamide, was known to suppress movement of the charcoal meal due to its anticholinergic, antihistaminic, and PG blocking effects [50]. Owing to its importance in the activation of smooth muscles contractions, Ca^2+^ has been mentioned associated with antidiarrheal effect of different drugs. Therefore, *C. aurea* could reduce the distance travelled by charcoal meal through decreasing intracellular Ca^2+^ level.

This study further reveals that the graded doses of both extracts significantly reduced intestinal motility in castor oil-induced intestinal transit model as compared to the negative controls. Drugs with antidiarrheal effects are well known for reducing gastrointestinal contractions and thereby slows the intestinal transit [51], allowing more time for better absorption of water and electrolytes [52]. In this regard, inhibition of diarrhea observed in this study might be due to the extracts' intestinal motility reducing ability. The reduction in intestinal transit seems comparable for both extracts, which could be credited to the presence of comparable level of secondary metabolites. Antidiarrheal properties of the plant extracts might be due to phytochemical constituents such as polyphenols, flavones, alkaloids, saponins, and terpenoids that are previously detected in the 80% methanol extract of *C. aurea* seeds [53]. Flavonoids are known to inhibit intestinal motility [17] through relaxing intestinal smooth muscles [54]. Terpenoids, on the other hand, were reported to inhibit intestinal motility and secretion by inhibiting the release of PGs [27].

By using the ADI value, which is a measure of the augmented effects of different components of diarrhea such as reduction in gastrointestinal motility, onset of diarrhea, and intestinal fluid accumulation, it is possible to have an overall understanding of antidiarrheal effect of the plant extract [55]. A higher the ADI value suggests that the plant extract is good in curing diarrhea [18]. In the current study, the ADI value indicated that both extracts of the plant have comparable diarrheal inhibition effect with loperamide at the highest dose tested. The study tested the antidiarrheal effects of *Mimusops kummel* fruit extract on mice also confirmed that the greatest antidiarrheal effect was obtained at 400 mg/kg, that is the highest dose [56].

The aqueous and 80% methanol seed extracts of the *C. aurea* were found to have marginal antibacterial activity on most of the tested organisms except *P. aeruginosa*. Considering MIC and MBC, better effect seemed to be observed against *S. typhimurium*, as its growth was inhibited at a concentration of 250 mg/ml and completely killed at a concentration of 500 mg/ml with 80% methanol seed extract of *C. aurea*. In terms of zone of inhibition, however, *E. coli* showed better response. The difference may be due to difference in sensitivity of the organisms to the plant extract, i.e., the 80% methanol seed extract of *C. aurea* may be bactericidal against *S. typhimurium* and bacteriostatic against *E. coli.* In general, antibacterial effect is insignificant. Even doses as high as 125 mg/ml did not produce the required zone of inhibition (>16 cm) to say the extract is effective. The activity of *C. aurea* seed extract at the high concentration of 1000 and 500 mg/ml may be due to toxicity of 80% methanol extract.

Although the plant is blessed with all of previously mentioned activities, it is not free from toxicity. Intoxication level is reliant on the dose of the plant extract administered, the higher the dose the more severe the toxicity. A study assessing antimalarial activity of *C. aurea* leaf extracts supports this finding [15]. Another research by Umer et al. [6] that assessed the antidiarrheal activity of 80% methanol leaf extract of *C. aurea,* however, reported that this plant is safe even up to 5000 mg/kg dose. The difference might be due to difference in plant parts tested and environmental factors from where the plant is taken. It is secondary plant metabolites that are mostly associated with toxicity. Plant poisoning most frequently comes from secondary metabolites such as furanocoumarins, alkaloids, and cyanogenic glycosides [57]. A preliminary study that screened the phytochemical constituents of *C. aurea* seeds confirmed that alkaloids and glycosides are parts of the plant's secondary metabolites [18]. Among the alkaloids that are known to pose a detrimental health impact to humans, the pyrrolizidine subtypes are the most reported one. Three plant families (Asteraceae, Fabaceae, and Boraginaceae) are the rich sources of pyrrolizidine alkaloids [17]. Photochemistry of *C. aurea* studied by Asres and his colleagues [58] further supported that the plant is endowed with many types of alkaloids, including pyrrolizidine alkaloids. In this regard, *C. aurea*, which is belonging to the family of Fabaceae and confirmed to have pyrrolizidine alkaloids, could potentially be toxic and judicious use is warranted.

## 5. Conclusion

Finding of this study revealed that both extracts of *C. aurea* have beneficial effect in controlling diarrhea through antisecretory and antimotility activities, lending support to the use of the plant as antidiarrheal remedy. Both extracts had comparable activity, indicating that medium polar to polar constituents contributing to the observed effect. Antibacterial effect is, however, insignificant and not promising to use as antibacterial agent to treat infectious diarrhea.

## Figures and Tables

**Table 1:** Effect of the aqueous and 80% methanol seed extracts of *C. aurea* on castor oil-induced diarrhea model in mice.

Groups	Onset of diarrhea	Total stool frequency in 4 hrs	Total weight of wet diarrhea	% Inhibition of total wet fecal output	Weight of watery content of wet stools	% Inhibition of watery content of wet stool
DW	79.83 ± 2.78	9.17 ± 1.11	1.29 ± 0.08	—	0.69 ± 0.11	—
L3	147.00 ± 2.89^a1^	2.33 ± 0.21^a3^	0.56 ± 0.09^a2^	56.59%	0.14 ± 0.03^a1^	79.71%
CAW60	151.67 ± 19.23^a2^	3.17 ± 0.75^a3^	0.82 ± 0.21	36.43%	0.38 ± 0.18	44.93%
CAW120	156.00 ± 22.27^a2^	2.17 ± 0.54^a3^	0.65 ± 0.17^a1^	49.61%	0.24 ± 0.11	65.23%
CAW240	177.83 ± 7.67^a3^	1.83 ± 0.31^a3^	0.48 ± 0.07^a2^	62.80%	0.11 ± 0.03^a2^	84.06
CAM60	103.17 ± 10.08	2.5 ± 0.34^a3^	0.81 ± 0.15^a1^	37.21%	0.32 ± 0.13^a1^	53.62%
CAM120	126.33 ± 20.73	1.67 ± 0.21^a3^	0.54 ± 0.11^a3^	58.14%	0.18 ± 0.05^a2^	73.91%
CAM240	203.67 ± 20.77^a3^	1.17 ± 0.17^a3^	0.38 ± 0.05^a3^	70.54%	0.11 ± 0.01^a3^	84.06%

Values are expressed as mean ± S.E.M (*n* = 6), analysis was performed using one way ANOVA followed by Tuckey post hoc test. Comparison was made among different groups: ^a^compared to control; ^1^*p* < 0.05, ^2^*p* < 0.01, ^3^*p* < 0.001. DW = distilled water, L = loperamide, CAW = *C. aurea* water extract, and CAM = *C. aurea* 80% methanol extract.

**Table 2:** Effects of the aqueous and 80% methanol seed extracts of *C. aurea* on castor oil-induced gastrointestinal transit in mice.

Group	Total length of small intestine (cm)	Distance moved by the charcoal meal (cm)	Peristalsis index (%)	%Inhibition
DW	56.00 ± 1.13	44.17 ± 1.33	79.00 ± 2.63	—
L3	53.83 ± 1.28	16.67 ± 1.33^a3c3^	30.81 ± 1.96^a3c3^	62.26%
CAW60	52.67 ± 0.88	30.67 ± 1.98a3b3^d3e3^	58.29 ± 3.74^a3b3d3e3^	30.22%
CAW120	51.50 ± 1.45	18.83 ± 2.34^a3c3^	36.63 ± 4.52^a3c3^	57.37%
CAW240	54.00 ± 1.32	16.17 ± 1.05^a3c3^	29.82 ± 1.31^a3c3^	63.39%
CAM60	52.00 ± 1.81	22.17 ± 2.73^a3^	42.23 ± 3.93^a3e1^	49.81%
CAM120	55.67 ± 1.17	18.83 ± 2.47^a3^	34.03 ± 4.69^a3^	57.37%
CAM240	59.83 ± 1.05	17.17 ± 1.45^a3^	28.55 ± 2.08^a3c1^	61.13%

Values are expressed as mean ± S.E.M (*n* = 6), analysis was performed using one way ANOVA followed by Tuckey post hoc test, comparison was made among different groups: ^a^compared to control, ^b^ to standard drug, ^c^ to 60 mg/kg aqueous, ^d^ to 120 mg/kg aqueous, ^e^ to 240 mg/kg aqueous; ^1^*p* < 0.05, ^2^*p* < 0.01, ^3^*p* < 0.001. DW = distilled water, L = loperamide, CAW = *C. aurea* water extract, and CAM = *C. aurea* 80% methanol extract.

**Table 3:** Effects of the aqueous and 80% methanol seed extracts of *C. aurea* on normal gastrointestinal transit in mice.

Group	Total length of small intestine (cm)	Distance moved by the charcoal meal (cm)	Percent of transit inhibition	%Inhibition
DW	52.83 ± 1.76	35.50 ± 0.99	32.38 ± 3.31	—
L3	54.00 ± 0.97	25.00 ± 2.88^a1^	53.70 ± 5.14^a1c1^	29.58%
CAW60	51.67 ± 1.52	31.83 ± 3.74	32.58 ± 7.93^b1^	10.34%
CAW120	51.00 ± 2.45	26.67 ± 1.45	47.65 ± 2.01	24.87%
CAW240	53.33 ± 0.95	26.50 ± 1.38	50.17 ± 2.90	25.35%
CAM60	50.83 ± 1.40	31.00 ± 3.48	38.68 ± 7.01	12.68%
CAM120	52.17 ± 0.70	26.83 ± 2.96	48.36 ± 5.94	24.42%
CAM240	53.83 ± 2.51	25.33 ± 2.64	51.70 ± 6.32	28.65%

Values are expressed as Mean ± S.E.M (*n* = 6), analysis was performed using one way ANOVA followed by Tuckey post-hoc test, Comparison was made among different groups: ^a^ compared to control, ^b^ to standard drug, ^c^ to 60 mg/kg aqueous, ^1^*p* < 0.05, ^2^*p* < 0.01, ^3^*p* < 0.001. DW = distilled water, L = loperamide, CAW = *C. aurea* water extract and CAM = *C. aurea* 80% methanol extract.

**Table 4:** *In Vivo* antidiarrhea index of aqueous and 80% methanol seed extracts of *C. aurea.*

Extracts	Dose administered	Delay in defecation (time of onset in minute, dfreq (%))	Gut meal travel distance, (Gmeq (%))	Reduction in intestinal fluid accumulation (%)	Antidiarrheal index (ADI)
Aqueous extract	60 mg/kg	47.37	30.22	33.96	36.50
	120 mg/kg	48.83	57.37	50.94	52.26
	240 mg/kg	55.11	63.39	67.92	61.91

80% methanol extract	60 mg/kg	22.62	49.81	39.62	35.47
	120 mg/kg	36.81	57.37	52.83	48.14
	240 mg/kg	60.80	61.13	62.26	61.39

Loperamide	3 mg/kg	45.69	62.26	49.06	51.87

Values are expressed as % inhibition of different parameters of different models and the combined effect is calculated as ADI.

**Table 5:** Antibacterial effects of aqueous and 80% methanol seed extracts of *C. aurea* using disk diffusion techniques.

	Zone of inhibition				
	Name of bacterial strain				
Category of test	Concentration	*E.coli* (ATCC25922)	*S.aureus* (ATCC25923)	*S.soni* (ATCC12022)	*S. typhimurium* (ATCC13331)
Methanol extract	1000 mg/ml	12.7 ± 0.17	11.57 ± 0.09	9.50 ± 0.18	9.20 ± 0.06
	500 mg/ml	11.54 ± 0.34	10.00 ± 0.06	—	8.70 ± 0.1
	250 mg/ml	9.9 ± 0.06	7.5 ± 0.01	—	8.00 ± 0.15
	125 mg/ml	9.51 ± 0.11	7.25 ± 0.13	—	7.34 ± 0.09
	Cipro5 *μ*g/disc^*∗*^	28 ± 0.15	26.00 ± 0.15	28.00 ± 0.17	27.00 ± 0.1

Aqueous extract	1000 mg/ml	—	8.73 ± 0.09	—	8.70 ± 0.15
	500 mg/ml	—	7.7 ± 0.02	—	7.30 ± 0.15
	250 mg/ml	—	6.48 ± 0.14	—	—
	125 mg/ml	—	—	—	—

Values are expressed as Mean ± S.E.M (*n* = 3). The negative control showed no antibacterial activity ^*∗*^ = positive control, cipro = ciprofloxacin, — = no activity, The values are the average of triplicate tests. ATCC—American type culture collection, E. *coli* = *Escherichia coli*, S. *aureus* = *Staphylococcus aureus*, *S*. *soni* = *Shigella soni*, *S. typhimurium* = *Salmonella typhimurium*.

**Table 6:** Antibacterial effects of both aqueous and 80% methanol seed extracts of *C. aurea* using microdilution techniques.

Minimum inhibitory concentration and minimum bactericidal concentration				
Organism	Methanol extract		Aqueous extract	
	MIC	MBC	MIC	MBC
*E. coli* (ATCC25922)	500 mg/ml	1000 mg/ml	—	—
*S. aureus* (ATCC25923)	500 mg/ml	1000 mg/ml	1000 mg/ml	—
*S. typhimurium* (ATCC13331)	250 mg/ml	500 mg/ml	500 mg/ml	1000 mg/ml
*S. soni* (ATCC12022)	1000 mg/ml	—	—	—

MIC—minimum inhibitory concentration, MBC—minimum bactericidal concentration, the values are the average of triplicate tests. ATCC—American type culture collection. — = no activity.

## Data Availability

All data used to support the findings of the study are included within the article.

## Abbreviations

ADI: Antidiarrheal Index
AIDS: Acquired immune deficiency syndrome
ANOVA: Analysis of variance
ATCC: American type culture collection
BHI: Brain heart infusion
cAMP: Cyclic adenosine monophosphate
CFU: Colony forming unit
cGMP: Cyclic guanosine monophosphate
ETEC: Enterotoxigenic *Escherichia coli*
GI: Gastrointestinal
IL: Interleukin
MBC: Minimum bactericidal concentration
MIC: Minimum inhibitory concentration
NO: Nitric oxide
OECD: Organization for economic cooperation development
PGs: Prostaglandins
RPM: Rotation per-minute.
